# X-Ray Repair Cross-Complementing Group 1 (XRCC1) Genetic Polymorphisms and Cervical Cancer Risk: A HuGE Systematic Review and Meta-Analysis

**DOI:** 10.1371/journal.pone.0044441

**Published:** 2012-09-12

**Authors:** Ya Li, Fei Liu, Shi-Qiao Tan, Yan Wang, Shang-Wei Li

**Affiliations:** 1 Division of Reproductive Medical Center, West China Second University Hospital of Sichuan University, Chengdu, Sichuan Province, People’s Republic of China; 2 Division of Liver Transplantation, Department of Liver and Vascular Surgery, West China Hospital of Sichuan University, Chengdu, Sichuan Province, People’s Republic of China; 3 Division of Reproductive Endocrinology and Infertility, West China Second University Hospital of Sichuan University, Chengdu, Sichuan Province, People’s Republic of China; Duke University Medical Center, United States of America

## Abstract

**Background:**

Previous studies investigating the association between X-ray repair cross-complementation group 1(XRCC1) polymorphisms and cervical cancer (CC) risk has provided inconsistent results. The aim of our study was to assess the association between the XRCC1 gene Arg399Gln, Arg194Trp, Arg280His polymorphisms and risk of CC.

**Methods:**

Two investigators independently searched the Medline, Embase, CNKI, and Chinese Biomedicine Databases for studies published before March 2011.Summary odds ratios (ORs) and 95% confidence intervals (CIs) for XRCC1 polymorphisms and CC were calculated in a fixed-effects model or a random-effects model when appropriate.

**Results:**

Ultimately, 9, 5 and 2 studies were found to be eligible for meta-analyses of Arg399Gln, Arg194Trp and Arg280His, respectively. Our analysis suggested that the variant genotypes of Arg194Trp were associated with a significantly increased CC risk (Trp/Trp vs Arg/Arg, OR = 2.21, 95% CI = 1.60–3.06; Arg/Trp vs Arg/Arg, OR = 1.23, 95% CI = 1.02–1.49; dominant model, OR = 1.36, 95% CI = 1.14–1.63; recessive model, OR = 2.06, 95% CI = 1.51–2.82). For Arg280His polymorphism, no obvious associations were found for all genetic models. For Arg399Gln polymorphism, also no obvious associations were found for all genetic models. In the subgroup analyses by ethnicity/country, a significantly increased risk was observed among Asian, especially among Chinese. To get more precise evidences, adjusted ORs (95%CI) by potential confounders (such as age, ethnicity or smoking, etc) were also calculated for XRCC1 Arg399Gln and Arg194Trp, however, the estimated pooled adjusted OR still did not change at all.

**Conclusion:**

This meta-analysis suggests that Arg194Trp polymorphism may be associated with CC risk, Arg399Gln polymorphism might be a low-penetrent risk factor for CC only in Asians, and there may be no association between Arg280His polymorphism and CC risk.

## Introduction

The X-ray repair cross-complementing group 1 (XRCC1) protein, which is encoded by the XRCC1 gene, is an important component of the base excision repair (BER) pathway. SNPs in 1 susceptible gene have been increasingly emphasized on the grounds that XRCC1 is considered a crucial scaffold protein closely associated with the base excision repair pathway [1], [2], which has been thought of as the predominant DNA-damage repair pathway for the processing of small base lesions derived from oxidation and alkylation damage [3]. The XRCC1 gene is located on chromosome 19q13.2–13.3 [4], spans a genetic distance of 33 kb, comprises of 17 exons and encodes a 70-kDa protein consisting of 633 amino acids [5]. Although there are more than 300 validated single nucleotide polymorphisms (SNPs) in the XRCC1 gene reported in the dbSNP database (http://www.ncbi.nlm.nih.gov/SNP), three of XRCC1 are most studied [6], [7] and lead to amino acid substitutions in XRCC1 at codon 194 (at position 26304 on exon 6, base C to T, amino acid Arg to Trp, dbSNP no. rs1799782), codon 280 (at position 27466 on exon 9, base G to A, amino acid Arg to His, dbSNP no. rs25489) and codon 399 (at position 28152 on exon 10, base G to A, amino acid Arg to Gln, dbSNP no.rs25487), these non-conservative amino acid changes may alter XRCC1 function. This change in protein biochemistry leads to the supposition that variant alleles may diminish repair kinetics, thereby influencing susceptibility to adverse health effects, including cancer.

Exposure to different endogenous and exogenous mutagens and carcinogens can result in various types of DNA damages. These alterations, if not repaired, can cause genetic instability, mutagenesis and cancer. Importantly, to counteract the deleterious consequences of the DNA-damaging agents, evolution has moulded a number of DNA repair systems that as a whole take care of most of the insults inflicted on a cell’s vital genetic information. The repairing of different types of DNA damages is important for safeguarding genomic integrity [8]. Among the main DNA maintenance mechanisms operating in humans, the BER is the primary defence against lesions generated by ionizing radiation and strong alkylating agents as well as lesions formed by endogenous DNA-damaging agents like viruses [9].

Cervical cancer(CC) is the second most common malignancy among women worldwide, and continues to be a leading cause of cancer death in women. In developing countries, where widespread screening is still unavailable, cervical cancer accounts for a disproportionate proportion of the mortality [10], [11]. The highest incidence rates are observed in sub-Saharan Africa, Melanesia, the Caribbean, South central and Southeast Asia and Latin America [12]. Various evidences showed a strong link between the development of cervical cancer and high-risk human papillomavirus (HR-HPV) infection [13], such as HPV 16, 18, 31, 33, 35, 39, 45, 51, 52, 56, 58, 59, 68, and others. However, most of HPV infections are transient and only a small fraction of women infected with HPV will develop CC [14]. This indicates that HPV infection is a necessary event but not sufficient for CC. Therefore, other factors, including environmental agents and host genetic background, may play crucial roles in the development of CC [15]. Identification of genetic variants associated with cervical cancer will contribute to the understanding of underlying mechanisms behind its development and potentially provide therapeutic targets.

Over the last two decades, a number of case–control studies [16]–[25] were conducted to investigate the association between XRCC1 Arg194Trp, Arg280His, Arg399Gln polymorphisms and risk of CC in women. But these studies reported conflicting results. Different methodologies have been used, but, in particular, some of the studies used a small sample size and it is therefore not surprising that there has been a lack of replication in the various studies. By using all the available published data to increase the statistical power, it was hypothesized that a meta-analysis might allow plausible candidate genes to be excluded and causative genes to be identified with reliability. We have therefore taken a meta-analysis in which all the published case-control studies are processed to confirm whether the Arg194Trp, Arg280His, Arg399Gln polymorphism of XRCC1 gene promoter increased the risk of CC.

## Materials and Methods

### Search Strategy

PubMed, EMBASE, CNKI (China National Knowledge Infrastructure) and Chinese Biomedicine databases (the last search was updated in March 2011) were used simultaneously with the combination of the English and/or Chinese key terms: ‘X-ray repair cross -complementing group 1′ or ‘XRCC1’, or ‘BER’, ‘polymorphism’ or ‘genotype’ or ‘allele’, and ‘cervical cancer’ or ‘carcinoma of cervix’ or ‘cervical carcinoma’. All published papers in English language and Chinese language with available full text matching the eligible criteria were retrieved. In addition, we also checked the references of relevant reviews and eligible articles that our search retrieved. If more than one article was published by the same author using the same case series, we selected the study where the most individuals were investigated.

### Selection Criteria and Identification of Studies

For inclusion in this meta-analysis, the identified articles had to provide information on the following: (i) XRCC1 Arg194Trp, Arg280His or Arg399Gln polymorphisms and CC risk (Regardless of squamous cell carcinoma or adenocarcinoma), (ii) using a case–control or cohort design; (iii) sufficient data for examining an odds ratio (OR) with 95% confidence interval (CI); (iv) the most recent and/or the largest study with extractable data should be included concerning studies with overlapping patients and the controls. Major reasons for the exclusion of studies were as follows: (i) duplicate data, (ii) abstract, comment, review and editorial and (iii) no sufficient data were reported.

### Data Extraction

Two investigators (Ya Li and Fei Liu) extracted information from all eligible publications independently according to the inclusion criteria listed above. Disagreements were resolved by discussion between the two investigators. If the two authors could not reach a consensus, then a third investigator (Shang-Wei Li) was consulted to resolve the dispute and a final decision was made by the majority of the votes. The following characteristics were collected from each study: first author, year of publication, country/region of the first or corresponding author, ethnicity, number of cases and controls, genotyping methods, minor allele frequency (MAF) in controls, and evidence of Hardy–Weinberg equilibrium (HWE). Different ethnicities were categorized as Asian, Caucasian. If original genotype frequency data were unavailable in relevant articles, a request was sent to the corresponding author for additional data.

### Statistical Analysis

We first assessed HWE in the controls for each study using goodness-of-fit test (*chi-square* or *Fisher’s exact* test) and a *P*<0.05 was considered as significant disequilibrium. The strength of the association between CC and the XRCC1 Arg194Trp, Arg280His and Arg399Gln polymorphisms were estimated using ORs, with the corresponding 95% CIs. The significance of pooled ORs was tested by Z test (*P*<0.05 was considered significant). For XRCC1 Arg194Trp polymorphism, we first examined the risk of the variant genotypes Trp/Trp or Arg/Trp on CC compared with the wild-type Arg/Arg homozygote. Then, the risk of (Trp/Trp + Arg/Trp) vs. Arg/Arg and Trp/Trp vs (Arg/Trp + Arg/Arg) for CC was evaluated in dominant and recessive models. For XRCC1 Arg280His and Arg399Gln polymorphisms, we also performed the four genetic models. If feasible, we also carried out the stratified analyses by ethnicity, country, publication time, study sample size.

Both the Cochran’s Q statistic [26] to test for heterogeneity and the *I* ^2^ statistic to quantify the proportion of the total variation due to heterogeneity [27] were calculated. A *P* value of more than the nominal level of 0.10 for the Q statistic indicated a lack of heterogeneity across studies, allowing for the use of a fixed-effects model (the Mantel–Haenszel method) [28]; otherwise, the random-effects model (the DerSimonian and Laird method) was used [29]. To explore sources of heterogeneity across studies, we did logistic meta-regression analyses. We examined the following study characteristics: ethnicity, country, HWE in controls (yes/no), genotyping methods and study sample size (≤400 and >400 subjects). Sensitivity analysis was performed to assess the stability of results. Cumulative meta-analyses of associations for each SNP were also conducted through assortment of studies with publication time.

Several methods were used to assess the potential publication bias. Visual inspection of funnel plot asymmetry was conducted. The Begg’s rank correlation method [30] and the Egger’s weighted regression method [31] were used to statistically assess publication bias (*P*<0.05 was considered statistically significant). All statistical analyses were performed with the Stata software (version 11.0; STATA Corp., College Station, TX, USA) using two-sided *P*-values.

## Results

### Literature Search and Study Selection

**Figure 1:**
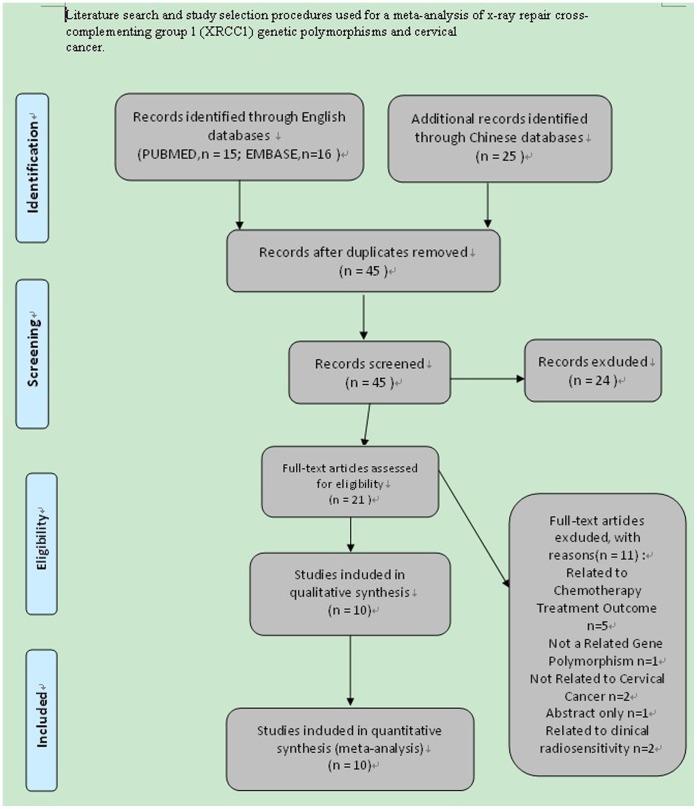
Literature search and study selection procedures used for a meta-analysis of x-ray repair cross-complementing group 1 (XRCC1) genetic polymorphisms and cervical cancer.

56 papers were relevant to the search words. Through screening the title and reading the abstract and the entire article,10 eligible articles [16]–[25] (six [16]–[21] in English and four [22]–[25] in Chinese) were included based on the search criteria, one of which were the dissertations of postgraduate students [24], for CC susceptibility related to the XRCC1 gene Arg194Trp, Arg280His and Arg399Gln polymorphisms. The literature search and study selection procedures are shown in Figure 1.

Study characteristics were summarized in Table 1. There were seven studies of subjects of Asian descent, two study of subjects of Caucasian descent and one of subjects Latin America descent. Among these studies, 5 studies have investigated only XRCC1 Arg399Gln polymorphism, 3 studies included XRCC1 Arg194Trp and Arg399Gln polymorphisms, whereas 1 studies included XRCC1 Arg194Trp, Arg280His and Arg399Gln polymorphisms, and 1 study included XRCC1 Arg194Trp and Arg280His polymorphisms. Therefore, there were 9 case-control studies with 1761 cases and 2552 controls for Arg399Gln polymorphism, 5 case-control studies with 893 cases and 1237 controls for Arg194Trp polymorphism and 2 case-control studies with 662 cases and 975 controls for Arg280His polymorphism. Studies had been carried out in China, Japan, Slovakia, Poland, Thailand and Argentina. The controls were mainly from healthy population or blood donor. 9/10 studies extracted DNA from peripheral blood and a classic PCR-RFLP assay was used in 8 out of 10 studies. Only 5/10 (50%) studies described the use of positive controls and a different genotyping assay to confirm the data. The genotype distributions among the controls of all studies followed HWE except for two studies [19], [20] for the Arg194Trp polymorphism and one study [22] for the Arg280His polymorphism.

**Table 1:** Characteristics of studies included in this meta-analysis.

**Gene**	**Author**	**Year**	**Ethnicity**	**Country**	**Sample Size**		**Genotyping**	**Matching Criteria**	**MAF in**		**HWE**
**Polymorphism**	**Reference**				**Case**	**Control**	**Methods**		**Case**	**Control**	
Arg194Trp	Huang [18]	2007	Asian	China	539	800	MA-PCR	Age,resident and medical history	0.35	0.29	Yes
	Farkasova [19]	2008	Caucasian	Slovakia	17	30	PCR-RFLP	_	_	_	_
	Wang [23]	2010	Asian	China	123	175	PCR-RFLP	Age and medical history	0.28	0.19	Yes
	Settheetham-Ishida [20]	2011	Asian	Thailand	111	118	PCR-RFLP	Medicalhistory	0.30	0.23	No
	Barbisan [21]	2011	Latin America	Argentina	103	114	PCR-RFLP	Age	0.14	0.09	No
Arg280His	Huang [18]	2007	Asian	China	539	800	MA-PCR	Age, residentand medicalhistory	0.12	0.12	Yes
	Wang [23]	2010	Asian	China	123	175	PCR-RFLP	Age andmedical history	0.14	0.10	No
Arg399Glu	Niwa [17]	2005	Asian	Japan	131	320	PCR-RFLP	Age, medical history and smoking	0.29	0.25	Yes
	Huang [18]	2007	Asian	China	539	800	MA-PCR	Age, residentand medicalhistory	0.28	0.19	Yes
	Hong [25]	2008	Asian	China	72	176	Taqman	Age, race, relationship, medical history and sex history	0.26	0.22	Yes
	Farkasova [19]	2008	Caucasian	Slovakia	18	30	PCR-RFLP	_	_	_	_
	Jiang [24]	2009	Asian	China	436	503	PCR-RFLP	Medical history	0.27	0.27	Yes
	Xiao [26]	2010	Asian	China	162	183	PCR-RFLP	Age and medical history	0.27	0.30	Yes
	Settheetham-Ishida [20]	2011	Asian	Thailand	111	118	PCR-RFLP	Medical history	0.22	0.23	Yes
	Barbisan [21]	2011	Latin America	Argentina	103	114	PCR-RFLP	Age	0.33	0.42	Yes
	Roszak [22]	2011	Caucasian	Poland	189	308	PCR-RFLP	Age	0.47	0.37	Yes

PCR-RFLP, polymerase chain reaction - restriction fragment length polymorphism; MA-PCR, mismatch amplification - polymerase chain reaction; MAF, minor allele frequency; HWE, Hardy– Weinberg equilibrium in controls.

### XRCC1 Arg194Trp and Arg280His Polymorphism

**Figure 2:**
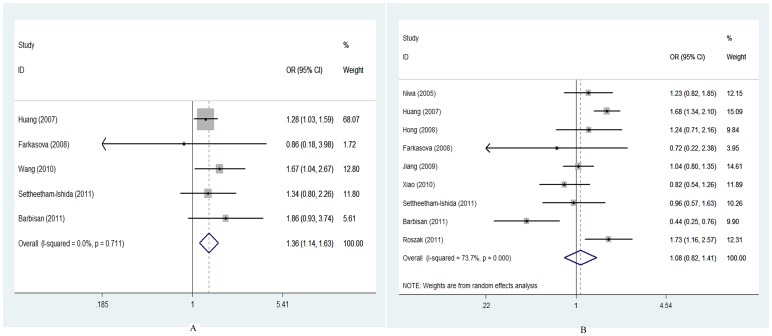
Forest plots of ORs with 95% CI for XRCC1 polymorphisms(Arg194Trp, Arg399Gln) and risk for cervical cancer. The center of each square represents the OR, the area of the square is the number of sample and thus the weight used in the meta-analysis, and the horizontal line indicates the 95%CI. (A) Arg194Trp, Trp/Trp+Trp/Arg vs. Arg/Arg. (B) Arg399Gln, Gln/Gln+Gln/Arg vs. Arg/Arg.

Five case-control studies [17]–[20], [22] with 893 cases and 1237 controls for XRCC1 Arg194Trp were included eventually. There was a wide variation in the XRCC1 Arg194Trp Trp allele frequency among different ethnicities, ranging from 9% in a Latin-America population [20] to 29% in an Asian population [17]. For the XRCC1 Arg194Trp polymorphism, a significantly increased CC risk was found when all studies were pooled into the meta-analysis (TrpTrp vs. ArgArg: OR = 2.21, 95% CI = 1.60–3.60, *P*_heterogeneity_ = 0.53; ArgTrp vs. ArgArg: OR = 1.23, 95% CI = 1.02–1.49, *P*_heterogeneity_ = 0.39; dominant model: OR = 1.36, 95% CI = 1.14–1.63, *P*_heterogeneity_ = 0.71; and recessive model: OR = 2.06, 95% CI = 1.51–2.82, *P*_heterogeneity_ = 0.54) (Figure 2A). When stratifying by ethnicity, a significantly increased risk was observed among Asian (TrpTrp vs. ArgArg: OR = 2.29, 95% CI = 1.63–3.20, *P*_heterogeneity_ = 0.46; dominant model: OR = 1.34, 95% CI = 1.11–1.62, *P*_heterogeneity_ = 0.61). Moreover, when subgroup analyses for studies with genotype distribution of controls in HWE or out of HWE, a significantly elevated risk was found among studies with genotype distribution of controls in HWE (TrpTrp vs. ArgArg, OR = 2.16, 95% CI = 1.53–3.05, *P*_heterogeneity_ = 0.61; dominant model: OR = 1.33, 95% CI = 1.09–1.62, *P*_heterogeneity_ = 0.52).

There were only two case–control studies [17], [22] which had been performed to study the XRCC1 Arg280His polymorphisms and CC risk. The results of the combined analyses showed that XRCC1 Arg280His polymorphism was not associated with CC risk (Table 2).

**Table 2:** The XRCC1 Arg194Trp and Arg280His polymorphisms on CC risk.

Variables	N^a^	Subgroup	OR (95%CI)	Pooled methods		*P* b–heterogeneity *P value*		*P*–Publication bias	
								Begg^c^	Egger^d^
Arg194Trp C/T									
Trp/Trp vs Arg/Arg	5	All	**2.21(1.60,3.06)**		Fixed M-H method	0.53	0.000	0.73	0.64
Arg/Trp vs Arg/Arg	5	All	**1.23(1.02,1.49)**		Fixed M-H method	0.39	0.03	0.31	0.15
Dominant	5	All	**1.36(1.14,1.63)**		Fixed M-H method	0.71	0.001	1.00	0.63
Recessive	5	All	**2.06(1.51,2.82)**		Fixed M-H method	0.54	0.000	0.73	0.73
Arg280His G/A									
His/His vs Arg/Arg	2	All(Asian)	1.60(0.74,3.44)		Fixed M-H method	0.18	0.23	1.00	NA
Arg/His vs Arg/Arg	2	All(Asian)	1.03(0.81,1.32)		Fixed M-H method	0.82	0.81	1.00	NA
Dominant	2	All(Asian)	1.07(0.84,1.36)		Fixed M-H method	0.37	0.58	1.00	NA
Recessive	2	All(Asian)	1.59(0.74,3.41)		Fixed M-H method	0.18	0.24	1.00	NA

NA, not application.

a Number of comparisons.

b *P* value of Q-test for heterogeneity test. Random-effects model was used when *P* value for heterogeneity test <0.1; otherwise, fixed-effects model was used.

c *P* of Begg, *P* value of Begg rank correlation method for testing publication bias.

d *P* of Egger, *P* value of Egger rank correlation method for testing publication bias.

### XRCC1 Arg399Gln Polymorphism

Nine case-control studies [16]–[21], [23]–[25] with 1761 cases and 2552 controls were included for association between XRCC1 Arg399Gln polymorphism and CC risk. There was a wide variation in the XRCC1 Arg399Gln Gln allele frequency among different ethnicities, ranging from 19% in an Asian population [17] to 42% in a Latin-America population [20].The genotype distributions among the controls of all studies were consistent with HWE for the XRCC1 Arg399Gln polymorphism.

The evaluations of the association of XRCC1 Arg399Gln polymorphism with CC risk are shown in Table 3. The results of the combined analyses showed that XRCC1 Arg399Gln was not associated with CC risk for all genetic models (GlnGln vs. ArgArg: OR = 1.20, 95% CI = 0.78–1.84, *P*_heterogeneity_ = 0.003; ArgGln vs. ArgArg: OR = 1.07, 95% CI = 0.82–1.41, *P*_heterogeneity_ = 0.001; dominant model: OR = 1.08, 95% CI = 0.82–1.41, *P*_heterogeneity_<0.001; and recessive model: OR = 1.19, 95% CI = 0.86–1.66, *P*_heterogeneity_ = 0.06) (Figure 2B). In the subgroup analyses by ethnicity/country, a significantly increased risk was observed among Asian (ArgGln vs. ArgArg: OR = 1.24, 95% CI = 1.07–1.43, *P*_heterogeneity_ = 0.16), especially among Chinese (ArgGln vs. ArgArg: OR = 1.27, 95% CI = 1.08–1.49, *P*_heterogeneity_ = 0.07). When stratifying by study sample size, a significantly increased CC risk was observed among large sample studies (>400 subjects) (Arg/Gln vs. ArgArg: OR = 1.36, 95% CI = 1.17–1.59; dominant model: OR = 1.38, 95% CI = 1.06–1.81), but not among small sample studies (≤400 subjects). Interestingly, when stratifying by publication time, a significantly elevated risk was found among studies published before or during 2009(ArgGln vs. ArgArg: OR = 1.32, 95% CI = 1.13–1.55, *P*_heterogeneity = _0.26), but not among studies published after 2009.

**Table 3:** Stratified analyses of the XRCC1 polymorphism Arg399Gln on cervical cancer (CC) risk.

Variables	N^a^	Gln/Gln versus Arg/Arg		Arg/Gln versus Arg/Arg		Dominant model		Recessive model	
		OR(95%CI)	*P* b	OR(95%CI)	*P* b	OR(95%CI)	*P* b	OR(95%CI)	*P* b
Total	9	1.20(0.78,1.84)	0.003	1.07(0.82,1.41)	0.001	1.08(0.82,1.41)	<0.001	1.19(0.86,1.66)	0.06
Ethnicity									
Asian	6	1.15(0.70,1.90)	0.01	**1.24(1.07,1.43)**	0.16	1.16(0.91,1.49)	0.02	1.09(0.71,1.69)	0.04
Caucasian	2	1.30(0.40,4.26)	0.01	0.77(0.18,3.25)	<0.001	0.84(0.30,2.35)	<0.001	1.52(1.00,2.26)	0.33
Country									
China	4	1.15(0.57,2.30)	0.003	**1.27(1.08,1.49)**	0.07	1.18(0.84,1.65)	0.006	1.08(0.59,1.99)	0.01
Others	5	1.26(0.69,2.30)	0.07	1.04(0.82,1.31)	0.001	0.96 (0.59,1.58)	0.002	1.40(1.00,1.95)	0.62
Study sample size									
>400	4	1.50(0.82,2.74)	0.003	**1.36(1.17,1.59)**	0.22	**1.38(1.06,1.81)**	0.03	1.31(0.79,2.18)	0.01
≤400	5	0.82(0.52,1.28)	0.70	0.78(0.49,1.26)	0.03	0.81(0.63,1.03)	0.11	0.99(0.64,1.51)	0.81
Publication time									
Before or during 2009	5	1.33(0.71,2.50)	0.009	**1.32(1.13,1.55)**	0.26	1.27(0.98,1.64)	0.06	1.21(0.68,2.14)	0.02
After 2009	4	1.05(0.52,2.11)	0.03	0.85(0.48,1.50)	0.001	0.90(0.51,1.56)	0.001	1.25(0.89,1.75)	0.27

a Number of comparisons.

b *P* value of Q-test for heterogeneity test. Random-effects model was used when *P* value for heterogeneity test <0.1; otherwise, fixed-effects model was used.

### Heterogeneity Analysis

There was heterogeneity among studies in overall comparisons and also subgroup analyses for the XRCC1 Arg399Gln polymorphism. To explore sources of heterogeneity across studies, we assessed all of the comparison models by ethnicity (Asian/Caucasian), country (China/other), publication time (before or during 2009/after 2009), genotyping methods (PCR-RFLP/other), or study sample size (>400 subjects/≤400 subjects) when necessary. As a result, study sample size (dominant model: *P* = 0.04), but not the ethnicity, country, genotyping methods or publication time, was found to contribute to substantial heterogeneity. Moreover, meta-regression analyses indicated that study sample size could explain 55.25% of the *τ* ^2^.

### Sensitivity Analysis

**Figure 3:**
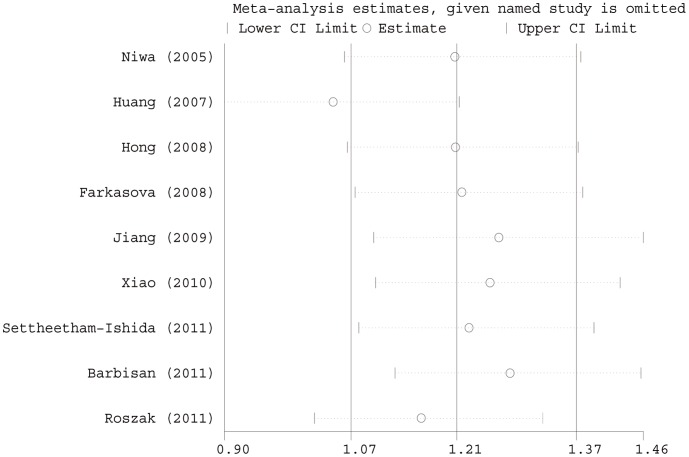
Influence analysis of the summary odds ratio coefficients on the association between x-ray repair cross-complementing group 1 gene (XRCC1) Arg399Gln GG-plus-GA genotypes with cervical cancer risk. Results were computed by omitting each study (left column) in turn. Bars, 95% confidence interval.

In the sensitivity analysis, the influence of each study on the pooled OR was examined by repeating the meta-analysis while omitting each study, one at a time. As for the association of the Arg399Gln SNP with CC risk, the study that had the most influence on the overall pooled estimates (Figure 3) seemed to be the one conducted by Huang *et al*. [18]; however, the sensitivity analysis showed that the ORs were 1.08 (95% CI: 0.82, 1.41) and 1.00 (95% CI: 0.77, 1.31) before and after the removal of that study, respectively, indicating high stability of the results. There were two studies which deviated from HWE for the XRCC1 Arg194Trp polymorphism, when excluding the studies that were not in HWE, the estimated pooled OR still did not change at all (Table 2).

The OR and 95%CI are adjusted for potential confounders (such as age, ethnicity or smoking, etc) in some studies, while the OR and 95%CI are not adjusted for these potential confounders in the other studies. The associations of these risk factors with cervical cancer are of magnitudes of at least similar range as the SNPs reported. When excluding the studies that were not adjusted for these potential confounders, the estimated pooled adjusted OR still did not change at all (Table 4). This procedure proved that our results were reliable and robust.

**Table 4:** Quantitative analyses for the relationships between XRCC1polymorphisms and cervical cancer(CC) risk based on adjusted OR (95%CI).

Genetic	Comparisons	Sample size		Adjusted OR	P_z-test_	P_heterogeneity_	Model
polymorphisms		N^a^	Case/Control	(95%CI)			
XRCC1 Arg399Gln	Gln/Gln vs. Arg/Arg	6		1.23(0.94,1.61)	0.127	0.012	Random
(Total)	Arg/Gln vs. Arg/Arg	6		1.04(0.74,1.45)	0.843	0.001	Random
	Gln/Gln+Arg/Gln vs. Arg/Arg	6		1.06(0.76,1.48)	0.736	0.000	Random
XRCC1 Arg399Gln	Gln/Gln vs. Arg/Arg	5		**1.34(1.01,1.78)**	0.046	0.017	Random
(Asian)	Arg/Gln vs. Arg/Arg	5		**1.29(1.11,1.50)**	0.001	0.276	Fixed
	Gln/Gln+Arg/Gln vs. Arg/Arg	5		1.25(0.98,1.59)	0.078	0.054	Random
XRCC1 Arg194Trp	Trp/Trp vs. Arg/Arg	3		**2.15(1.53,3.04)**	0.000	0.372	Fixed
(Total)	Arg/Trp vs. Arg/Arg	3		1.19(0.97,1.46)	0.106	0.338	Fixed
	Trp/Trp+Arg/Trp vs. Arg/Arg	3		**1.33(1.09,1.61)**	0.004	0.606	Fixed

### Cumulative Meta-analysis

**Figure 4:**
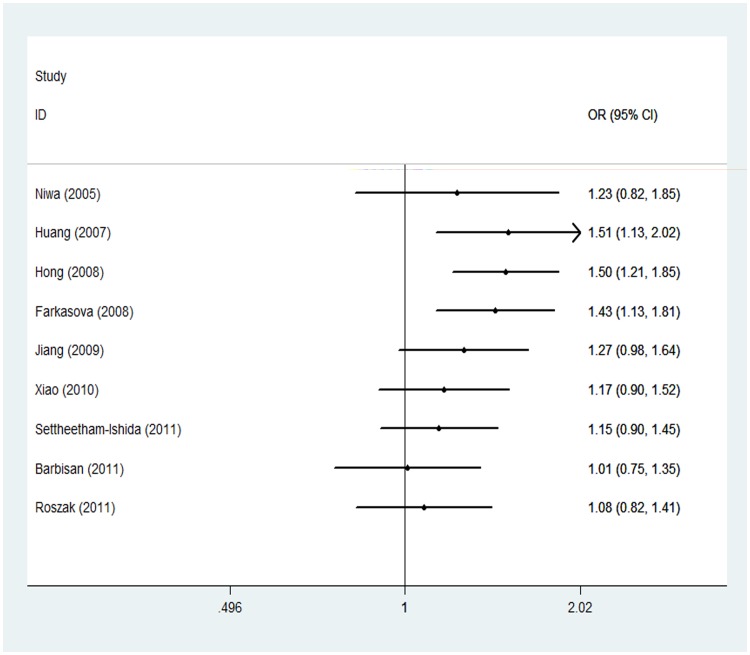
Results from cumulative meta-analysis of associations between x-ray repair cross-complementing group 1 gene (XRCC1) Arg399Gln GG-plus-GA genotypes (G carrier group), as compared with the AA genotype, and cervical cancer risk. The circles and horizontal lines show the accumulation of estimates as results from each study were added, rather than the estimate for each individual study. Studies sorted by publication time; Bars, 95% confidence interval.

Cumulative meta-analyses of the 3 associations were also conducted via the assortment of studies by publication time. Figure 4 shows results from the cumulative meta-analysis of the association of the Arg399Gln SNP with overall cervical cancer in chronologic order. Inclinations toward null significant associations were evident with each accumulation of more data over time, although associations were initially strong. Results for the other 2 SNPs are the same (data not shown).

### Publication Bias

**Figure 5:**
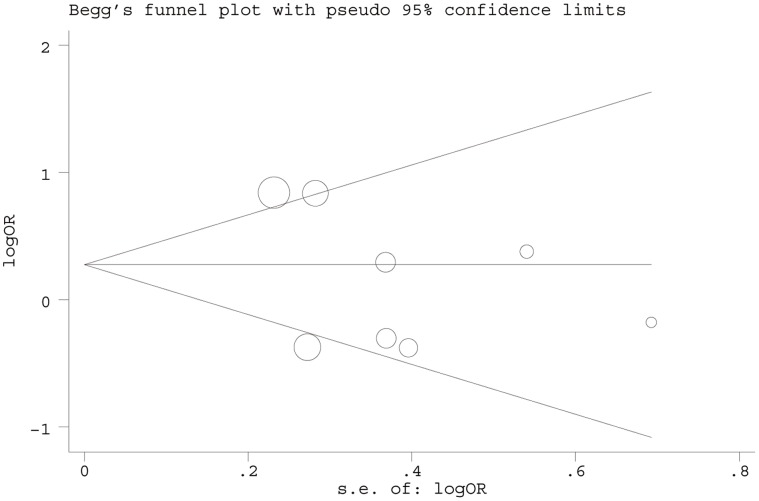
For XRCC1 Arg399Gln polymorphism, Begg’s funnel plot for publication bias test. (Gln/Gln vs. Arg/Arg). Each point represents a separate study for the indicated association. LogOR, natural logarithm of OR. Horizontal line, mean effect size.

Begg’s Funnel plot and Egger’s test were performed to evaluate publication bias of the literature on CC. Figure 5. displayed a funnel plot that examined the XRCC1 Arg399Gln polymorphism and overall CC risk included in the meta-analysis. The shape of funnel plot did not reveal any evidence of funnel plot asymmetry. The statistical results still did not show publication bias (for XRCC1 Arg194Trp and Arg280His polymorphism were in Table 2; and for XRCC1 Arg399Gln polymorphism: Gln/Gln vs. Arg/Arg: Begg’s test *P* = 0.54, Egger’s test *P* = 0.32; Arg/Gln vs. Arg/Arg: Begg’s test *P* = 0.06, Egger’s test *P* = 0.10; dominant model: Begg’s test *P* = 0.35, Egger’s test *P* = 0.14; recessive model: Begg’s test *P* = 0.39, Egger’s test *P* = 0.40).

## Discussion

Various DNA alterations can be caused by exposure to environmental and endogenous carcinogens. Most of these alterations, if not repaired, may result in genetic instability, mutagenesis and cell death. DNA repair mechanisms are important for maintaining genome integrity and preventing carcinogenesis. The XRCC1 protein is an important component of the BER pathway, which fixes base damage and DNA single-strand breaks caused by ionizing radiation and alkylating agents. Mutations of XRCC1 may increase the risk of cancers by impairing the interaction of XRCC1 with other enzymatic proteins and consequently altering DNA repair activity [32], [33]. In recent years, a large number of molecular epidemiological studies have been conducted to evaluate the role of polymorphisms in the DNA repair gene XRCC1 on CC risk; however, the results remain conflicting rather than conclusive.

Three polymorphisms in XRCC1 (Arg194Trp, Arg280His and Arg399Gln) have been frequently examined in the studies on cancer susceptibility. To the best of our knowledge, this is the first systematic review that has investigated the association of XRCC1 polymorphisms and CC risk. In this meta-analysis, we found that XRCC1 Arg194Trp polymorphism may be associated with CC risk, while XRCC1 Arg399Gln polymorphism might be a low-penetrent risk factor for CC only in Asian women, and there may be no association between Arg280His polymorphism and CC risk. The explanation for the results may be that functional variants in the XRCC1 gene may play a crucial role in the facilitation of human cancer development because of the alteration of BER functions [34]. Such as, the functional significance of XRCC1 Arg194Trp is mainly due to its location in an evolutionarily conserved linker region [35], and the XRCC1 Arg399Gln SNP may alter the efficiency of the repair process because of its location in the poly (ADP-ribose) poly-merase-binding domain [34], [36], [37]. The null association between XRCC1 Arg280His SNP and CC risk may be because there were only two studies with limited populations for the SNP in the analysis. Moreover, in the cumulative meta-analysis stratified by publication date, the tendency toward respective associations for the 3 SNPs could be spotted with each accumulation of more data over time.

Because the allele frequencies of polymorphisms and their effects on the cancer risk were diverse in the different ethnicities, we carried out subgroup analysis by ethnicity for the Arg194Trp and Arg399Gln SNPs. The results of combined analyses suggested that the Arg194Trp polymorphism was associated with an increased CC risk, while the XRCC1 Arg399Gln was not associated with CC risk when all the studies were pooled. However, when stratifying by ethnicity, a significantly increased risk was observed among Asian for the 2 SNPs. Studies on the association of XRCC1 polymorphisms with CC were predominantly conducted in Asian countries; only two were conducted in Western countries. Thus, possible ethnic differences in the association of XRCC1 polymorphisms with CC should be investigated further and confirmed as more studies are conducted in Western countries.

In the stratification analysis carried out according to the study sample size for the Arg399Gln SNP, a statistically significant finding was seen in the large sample group (>400 subjects) but not among small sample studies (≤400 subjects), which indicates that large sample studies may offer quite different outcomes than small sample studies. This is probably because studies with small sample size may have insufficient statistical power to detect a slight effect or may have generated a fluctuated risk estimate [38]. Thus, the use of a proper and large sample size study is very important in reducing biases in such genotype association studies. We strongly recommend that researchers design genetic polymorphism association studies with larger study sample size in the future.

In the present meta-analysis, we searched as many publications as we could. Most of the literature with full-text we searched are in English and Chinese, and we believe that most of the related literature have been obtained and screened in our study. Furthermore, one of the major concerns in a sound meta-analysis is the degree of heterogeneity that exists between the component studies; we carried out the Q-test and *I^2^* statistics to test the significance of heterogeneity. Obvious heterogeneity between studies was observed in overall comparisons and also some subgroup analyses for some models, and then meta-regression analysis was used to explore the sources of heterogeneity. We found that study sample size did contribute to potential heterogeneity. Another important issue for any meta-analysis is publication bias because of selective publication of reports. In the current study, Funnel plot, Begg’s and Egger’s tests were performed to evaluate this problem. Both the shape of funnel plots and the statistical results did not show publication bias.

Although we have put considerable effort and resources into testing possible association between XRCC1 gene polymorphisms and CC risk, there are still some limitations in this meta-analysis. First, we did not perform subgroup analysis by the pathological types of CC due to limited data in primary studies. Most of the CC were squamous cell carcinoma in the present study, however, a few CC were mixed by squamous cell carcinoma and adenocarcinoma. Because of different pathological types, subgroup analysis should be performed. However, only one study [16] in this meta-analysis reported separate genotype frequency for squamous cell carcinoma and adenocarcinoma, although several studies were mixed by squamous cell carcinoma and adenocarcinoma [16], [17], [21], which prevented us to perform this subgroup analysis. Second, gene–gene, and gene–environmental interactions were not addressed in this meta-analysis because of the lack of sufficient data. It is possible for specific environmental and lifestyle factors to alter those associations between gene polymorphisms and cancer risk. For example, Lao *et al*. [39] concluded that the Gln/Gln genotype of Arg399Gln was associated with a decreased risk of bladder cancer among ever smokers while the Arg399Gln polymorphism was not associated with bladder cancer risk in the total population. Thirdly, there was significant between-study heterogeneity from studies in overall comparisons and also subgroup analyses, and the genotype distribution in the control group also showed deviation from HWE in some studies. Last but not least, the number of studies and the number of subjects in the studies included in the meta-analysis were small, especially for Caucasians population and for Arg280His. Because of few papers included in our meta-analysis, resulting in the unstable association estimates, our results in relation to these polymorphisms should always be treated as preliminary, and additional meta-analyses with a large number of papers are necessary to validate the association in the future. In spite of these, our meta-analysis also had some advantages. First, we did not detect any publication bias indicating that the whole pooled result should be unbiased. What’s more, genetic meta-analysis was always performed without adjustment, due to limited data in primary studies. In this meta-analysis, besides quantitative analyses for all SNPs without adjustment, adjusted analyses by potential confounders (such as age, ethnicity or smoking, etc) were also performed for XRCC1 Arg399Gln and Arg194Trp polymorphisms. The results of adjusted analyses were persistent, which in turn confirmed the reliability of our meta-analysis.

In conclusion, the research of the relationship of XRCC1 polymorphisms and CC is very popular but conflicting at present. Our meta-analysis suggests that XRCC1 Arg194Trp polymorphism may be associated with CC risk, while XRCC1 Arg399Gln polymorphism might be a low-penetrent risk factor for CC only in Asian women, and there may be no association between Arg280His polymorphism and CC risk.
